# How much is a child worth? Providers’ and patients’ views and responses concerning ethical and policy challenges in paying for ART

**DOI:** 10.1371/journal.pone.0171939

**Published:** 2017-02-16

**Authors:** Robert Klitzman

**Affiliations:** Masters of Bioethics Program, Columbia University, New York, New York, United States of America; US Army Engineer Research and Development Center, UNITED STATES

## Abstract

Infertility treatments remain expensive and in many countries are covered by little, if any, insurance, raising critical questions concerning how patients and providers view and make decisions regarding these challenges. In-depth semi-structured interviews of approximately 1 hour were conducted with 37 IVF providers and 10 patients (17 physicians, 10 other providers and 10 patients), and were systematically analyzed. These data suggest current insurance policies and legislation pose critical ethical and logistical challenges for both patients and providers. These individuals face multiple uncertainties about costs and insurance, related to unclear causes of fertility, treatment length, costs and outcomes, and odds that insurers will cover expenses. Insurers frequently decline to agree to reimbursement beforehand, and decide only afterwards, case-by-case, generating stress. Patients and providers thus may not be able to predict how best to allocate limited resources. Providers may advocate for patients, but are usually unsuccessful. Patients may adopt several strategies: e.g., moving/seeking treatment elsewhere, switching or feeling “stuck” in jobs because of insurance, seeking “free” medications, going into debt, or using funds intended for other purposes. Patients do not perceive and respond to resource limitations as fixed phenomena—i.e., patients do not see treatment simply as “affordable” or not. Rather, patients face quandaries of how much to keep spending—how much a child is worth—and are forced to make complex risk/benefit calculations. Couples can disagree, straining relationships. In sum, these data, the first to explore how providers and patients struggle, view, and make decisions regarding limited insurance and resources for infertility, raise several critical ethical and policy issues. These data suggest that individuals have difficulty translating profoundly life-altering, deeply personal quests for meaning and fulfillment into purely economic terms. These findings thus have important implications for future policy, practice, research, and patient and provider education.

## Introduction

Increasing numbers of patients are seeking infertility treatments, which are relatively expensive and covered by little, if any, insurance, posing critical ethical and policy questions. Direct costs per couple range from approximately $13,000 in the US to over $4,000 in the United Kingdom (UK), Australia, Germany, and elsewhere [1] In 2011, in California, total cost for a successful delivery was $112,799 for in vitro fertilization (IVF) [2].

Countries differ widely in their laws and regulations concerning insurance coverage of Assisted Reproductive Technology (ART), but generally contain certain restraints. In the European Union in 2013, six countries provided complete coverage, three provided no coverage, and 15 provided partial coverage, which varied based on factors such as the woman’s age (e.g., only covering women under 40 years), marital status (only married heterosexual couples), or overall percent of costs (e.g., 40% of overall expenses)[3]. India provided no coverage, and Japan provided some [3]. The affordability of ART also varied across countries, with the cost for one cycle as a percent of the average annual disposable income of a single person with no dependent children, ranging from over 50% in the US and Mexico, to around 30% in Canada and around 20% in Switzerland and Portugal [1]. In one British study, 78% of patients paid for treatment themselves [4]. Many patients plan to use savings or retirement funds (49%), go into debt (32%) or refinance their home (12%); and 37% said cost influenced their treatment [4]. Financial constraints have been found to be the most common reason British patients stop treatment (46%), and 10% regretted not stopping treatment earlier [5] In one Turkish clinic, the leading cause for stopping treatment was depletion of finances for men (45.5%) but “unsuccessful treatments and fear of coping” for women (53.7%) [6]. Internationally, cost is the biggest source of anxiety for IVF patients with 82.6% [7] These cost constraints profoundly affect access and use of services. Wealthier patients (especially those with household incomes of more than $100,000 per year) and/or college education are more likely to use more ART services [8–11].

Debates have ensued over whether states should mandate increased insurance coverage, due to concerns that limited coverage incentivizes patients and providers to create more twins (or higher multiple births, such as triplets, though the incidence of these have been decreasing) [11]. Some observers have argued that insurance coverage for IVF should increase in order to decrease the incidence of twins [12], yet this proposed policy confronts several obstacles. In the US, the Affordable Care Act did not mandate infertility coverage as an “essential benefit” [13] currently only fifteen [14] states require that insurance companies provide some coverage for ART, and only eight states cover IVF, but the specific amounts and requirements range widely. States vary in whether all insurers must provider coverage or only HMOs, whether coverage includes particular procedures such as IVF, and whether the cause of infertility needs to be “medical” [15–17]. Partly due to state mandates, some insurers are increasingly covering limited amounts of ART. But many insurance companies continue to cover little, if any, of the cost. Insurers may pay for only limited amounts of services, and do so only for “medical infertility”–not disease prevention or “structural infertility” (i.e., single individuals or gay couples who wish to have children). ASRM advocates that insurance coverage be increased, since poorer patients are often unable to access care [18]. In recent years, several infertility clinics have offered patients financial “risk-sharing’ or refund programs, which offer patients “money-back” guarantees to provide certain outcomes; and ASRM has cautioned that such programs should fully inform patients about all relevant details, including costs, advantages, disadvantages, and realistic odds of success [19].

Yet, though research has shown that finances lead many patients to stop treatment, no studies have explored how patients actually view and make these decisions, and are affected by these stresses. Needs exist for evidence-based medicine informed by patient values; but various challenges may arise [20]. Rational decision making models, drawing on multiple criteria, have been applied regarding use of technology in many domains, such as the environment [21] and various aspects of health care [20]. Such modeling has been applied to one aspect of ARTs—hypothetical decisions, using strictly biological criteria, of whether to transfer one or more than one embryo into the uterus [22]. Yet, questions thus emerge concerning broader patient decisions of whether and how much ART as a whole to pursue, given the many personal factors and values entailed. Recent multidisciplinary research on declining fertility in the developed world has examined various possible determinants at the micro-level (socioeconomic and educational factors), meso-level (kinship systems) and macro-level (welfare policies) [23]. Yet desires for children can also be highly personal and subjective more than instrumental, making purely instrumental perspectives and approaches problematic [24]. Moreover, research on these determinants has focused on exploring these issues among fertile adults deciding on the number of children to have, rather than on infertile individuals wrestling with not only whether to have children, but how much to pay for necessary medical assistance, posing additional complexities and problems. Hence, important questions remain of whether current policies affect infertility providers and patients in other ways besides increasing the numbers of embryos transferred, and if so, how—e.g., how patients and providers are affected by, and make decisions regarding, these challenges—for instance, how prospective parents determine how much to pay for having a child, and how much is too much; how they choose whether to go into debt, and if so, by how much; whether patients differ in these decisions, and if so, how; and what psychological and other effects these challenges may have. These questions all have vital ethical and policy ramifications given ongoing debates concerning whether states should mandate that insurers cover more infertility treatment, and if so what, how much and for whom. Thus, as part of an in-depth qualitative interview study of ART providers and patients, exploring attitudes and experiences concerning several critical aspects of IVF (e.g., sex selection, upper age limits of prospective mothers, numbers of embryos implanted, reductions of multi-fetal pregnancies, and buying and selling eggs [25–29]), these issues concerning insurance and cost constraints were explored.

## Methods

Since no prior studies have been published examining how patients and providers viewed and made decisions about insurance and cost constrains of infertility treatment, qualitative methods were chosen because these can best elicit the full range and typologies of attitudes, interactions and practices involved, and can inform subsequent quantitative studies. Qualitative methods have long been established as vital in research in health care and other areas, and have been extensively demonstrated to provide critical insights that quantitative methods cannot [30–32]. These approaches have successfully revealed critical aspects of patient attitudes and practices concerning views of other topics related to IVF—e.g., patients’ decisions concerning disclosures of donor oocytes [33].

From a theoretical standpoint, Geertz [34] has advocated studying aspects of individuals’ lives, decisions, and social situations not by imposing theoretical structures, but by trying to understand the individuals’ own experiences, drawing on their own words and perspectives to obtain a “thick description.” The methods for the present study adapted elements from “Grounded Theory” [30], and were thus informed by techniques of “constant comparison,” with data from different contexts compared for similarities and differences, to see if they suggest hypotheses. This technique of generates new analytic categories and questions, and checks them for reasonableness. These methods have been used in several other studies on key aspects of health behavior and doctor-patient relationships and communications in genetics and other areas [35–39]. During the ongoing process of interviewing, the PI constantly considered how participants resemble or differ from each other, and the social, cultural, and medical contexts and factors that contribute to differences. Grounded Theory also involves both deductive and inductive thinking, building inductively from the data to an understanding of themes and patterns within the data, and deductively, drawing on frameworks from prior research and theories.

### Participants

In brief, as summarized on Table 1 and described more fully elsewhere [25–29], 37 in-depth semi-structured interviews of approximately 1 hour each were conducted with 27 ART providers– 17 physicians and 10 other providers (7 mental health providers, 2 nurses, and 1 other)–and 10 patients. One physician and three other providers were also themselves patients. Of the 10 patients, 7 were employed full-time in office settings, one was employed part-time, one had recently stopped working to become a graduate student, and one was unemployed (though her husband was employed full-time). Patients and providers were recruited through listservs, emails, and word-of-mouth. Providers were also recruited through national ASRM meetings (e.g., PGD and mental health provider interest group meetings of attendees who were not known to the PI (Principal Investigator) beforehand). The PI approached these meeting attendees to ascertain whether they might be interested in participating in an interview study, and if so, the PI subsequently emailed them information about it. Most of those asked agreed to participate, and did so. A mental health listserv was also used, which is received by approximately 60 members (not all of whom are active), of whom 15 responded, and the first 8 respondents were then interviewed. Additional interviews were conducted as background, for informational purposes, with 8 physicians, 9 mental health providers and 14 patients; and informed, but were not included in the final formal data analysis. Interviews for the formal data analyses were conducted with each group until “saturation” was reached (i.e., “the point at which no new information or themes are observed in the data” [40]). Interviewees were from across the United States. The Columbia University Department of Psychiatry Institutional Review Board approved the study. Interviewees received a detailed information sheet about the study, on the basis of which they gave consent verbally over the phone. The PI then recorded these participants’ verbal consent in response to the written information document. Interviews were conducted by phone since these individuals were located across the US. The IRB approved this consent procedure. Providers described interactions with multiple patients they had treated, and colleagues; and patients often described interactions with multiple providers and other patients.

**Table 1:** Characteristics of sample.

	Male	Female	Total
**PHYSICIANS**	14	3	**17**
Physicians who are also patients	0	1	1
**Type of Practice**			
University affiliated	5	1	6
Private Practice	9	2	11
**OTHER ART PROVIDERS (e.g., mental health providers, nurses)**	1	9	**10**
Other providers who are also patients	0	3	3
**PATIENTS**	1	9	**10**
**TOTAL**	16	21	**37**

### Instruments

The semi-structured interview questionnaire was drafted drawing on prior literature and explored patients’ and providers’ views, experiences and decisions concerning insurance and cost constraints and related issues regarding infertility treatment. Sample questions included:

- What challenges do you face in your work as an ART provider? How do you address these challenges?
- Have you faced challenges concerning cost constraints and insurance? If so, when and what? What did you do?
- How do you view these issues?
- How have your patients viewed and approached these issues?
- What additional thoughts do you have about these issues?

The full interview guide is available in S1 File.

### Data analysis

Transcriptions and initial analyses of interviews occurred during the period in which the interviews were being conducted, enhancing validity, and helped shape subsequent interviews. Once the full set of interviews was completed, subsequent analyses were conducted in two phases, primarily by trained research assistants (RAs) and the PI. In phase I, they independently examined a subset of interviews to assess factors that shaped participants’ experiences, identifying categories of recurrent themes and issues that were subsequently given codes. The PI and RAs read each interview, systematically coding blocks of text to assign “core” codes or categories—e.g., patient strategies for obtaining insurance; clinicians’ efforts to help patients with cost constraints. While reading the interviews, a topic name (or code) was inserted beside each excerpt of the interview to indicate the themes being discussed. The PI and RAs then worked together to reconcile these independently developed coding schemes into a single scheme. Next, a coding manual was prepared, defining each code and examining areas of disagreement until reaching consensus. New themes that did not fit into the original coding framework were discussed, and modifications made in the manual when deemed appropriate.

In phase II of the analysis, the PI and RAs independently content-analyzed the data to identify the principal subcategories, and ranges of variation within each of the core codes. They reconciled the sub-themes identified by each coder into a single set of “secondary” codes and an elaborated set of core codes. These codes assess subcategories and other situational and social factors, and included, for instance; patients moving to obtain insurance coverage or seeking free treatment; and clinicians speaking to insurance companies on patients’ behalf or providing free samples of medications.

Codes and sub-codes were then used in analysis of all of the interviews. To ensure coding reliability, the PI and an RA analyzed all interviews. Where necessary, multiple codes were used. Similarities and differences were assessed between participants, examining categories that emerged, ranges of variation within categories, and variables that may be involved. Areas of disagreement were examined through closer analysis until consensus was reached. Regularly for consistency and accuracy in ratings was checked regularly by comparing earlier and later coded excerpts. Text from the interviews is presented below to allow readers to appreciate the richness of the data obtained.

## Results

**Fig 1:**
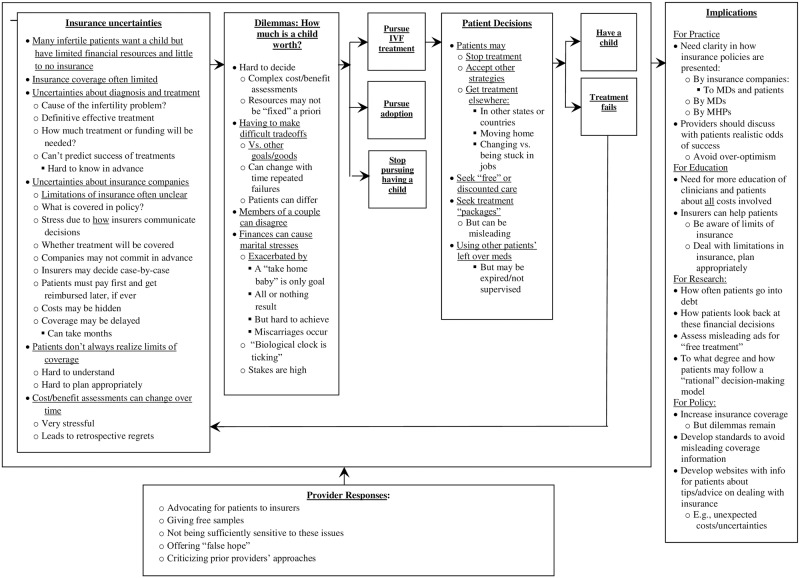
Challenges in paying for infertility treatment. (A) Insurance uncertainties; (B) Dilemmas: How much is a child worth?; (C) Pursue IVF treatment/Pursue adoption/Stop pursuing having a child; (D) Patient decisions; (E) Have a child/Treatment fails; (F) Implications; (G) Provider responses.

In brief, as described below and outlined in Fig 1, current insurer and governmental policies pose a series of ethical and logistical challenges for patients and providers, including multiple uncertainties concerning costs and coverage for ART—e.g., regarding the cause, length, prices, and outcomes of infertility treatments, and the likelihood that insurers will cover any particular procedure. Patients must hence often pay first, and later attempt to get reimbursed. But insurers frequently will not commit to coverage in advance, and instead make decisions only afterwards, on a case-by-case basis, which can take months. Patients and providers thus cannot predict in advance how best to allocate limited coverage amounts. Given these uncertainties, many patients adopt several strategies, and providers may seek to advocate for patients with insurers, but are usually unsuccessful. Patients, who continue to try to have a child, face quandaries of how much to keep spending—how much a child is worth—and must make complex risk/benefit decisions. A feedback loop exists, since most treatment cycles fail, and patients must then decide, after each failure, whether to try again and seek infertility treatment, or adoption, or not have a child.

### Uncertainties concerning insurance coverage

Patients face several different sets of uncertainties concerning possible insurance coverage for infertility—due to factors related both to themselves and their insurer.

Medical, legal and financial uncertainties arise regarding not only the extent of insurance coverage but, relatedly, the cause and treatment of infertility. These stresses prove to be inter-related.

#### Uncertainties concerning what kind of treatment will be needed and how much

Patients have difficulty planning in advance since they do not know beforehand the total costs—how long treatment may take. Vagaries arise for several reasons. First, the odds of any one cycle succeeding have been increasing over time, but may still only be approximately 40% [16]. Therefore, physicians cannot wholly predict the kind or amount of treatment that will be needed. Patients may not realize in advance that their insurance will not cover certain expenses. As one male patient, working part-time, described,

We asked, “What’s this going to cost? Can you tell us?” The doctor said: “I can’t guarantee anything. That’s with God—He does that. We just do the work and let him handle the rest.”

[Patient #2]

These high costs also exacerbate the stress of these uncertainties, and frustration. “It’s an awful lot of money for us to spend, and not have anything.” [Patient #2]

The fact that the future success of a procedure, once selected, cannot be wholly predicted, can generate uncertainty and regrets. Several factors, including the patient’s age and health, can affect the success of a procedure, but not in wholly foreseeable ways. One provider-patient’s doctor thought that intrauterine insemination (IUI) before IVF made sense (“Because my anatomy is so good and I had a pregnancy at 42, my doctor said, ‘I don’t think you’ll have any problems at all. I think you’ll need just a little bit.’” [Other Provider-patient #8]), but turned out to be wrong. Though providers must make judgment calls that may in the end prove incorrect, patients consequently cannot predict how many resources they will need.

Responses to any particular medication or procedure are also unpredictable, making it difficult to know how to allocate in advance limited resources effectively. As one provider-patient reflected,

I probably wouldn’t have utilized so much of my prescription coverage. The first time, I maxed out so much–$2,200 for prescriptions. I had 15 follicles. So the second time, we used half the medicine, and I had 8 follicles. I really didn’t need to blow up my ovaries, and would have had more medicine to do more IUIs.

[Other Provider-patient #8]

Hidden costs may also be involved. Insurance companies may cover some of the necessary diagnostic and therapeutic, but not other added expenses—e.g., paying for certain procedures, but not the meds, or vice versa. Patients may recognize unidentified extra costs only later and can rapidly add up. “The doctor talked about $15,000, just for one cycle. It didn’t actually include getting the sperm out of me. All they do is fertilize the egg.” [Patient #2]

#### Limitations of insurance not clear

Insurance companies may also be unclear upfront about what they will or will not cover, leaving patients uncertain, and exacerbating stress. In part, eligibility may depend on differing and unclear criteria. As one female patient, employed in an office, said,

For insurance companies to cover it, you had to match certain criteria. They told us that even for IVF, they would never cover 100%. So we were taking a risk, and might have to pay the whole thing on our own, because we were in that gray area. We weren’t considered infertile, because technically we could get pregnant on our own—we had gotten pregnant on our own the year before—but the trial and error part was very taxing emotionally and financially. That’s why we felt IVF was the better route for us. Technically, they should cover us, but you never really knew. Everybody on the phone said, “I can’t imagine it won’t go through; but I can’t say that for sure.” Insurance ended up covering everything.

[Patient #4]

Insurers may try to draw distinctions, covering certain “medical” causes of infertility, but not a wide range of other causes. Yet the etiology of a patient’s infertility may be unclear. Companies may cover certain medical conditions that happen to be linked to infertility problems.

Most insurance companies cover diagnostic procedures, and maybe some of the laboratory testing, but not hormonal testing and semen analysis. But surgery for specific problems such as testicular varicoceles, because it can adversely affect testicular growth, is also a health issue. I have operated on men with similar issues who were not trying to get pregnant, to preserve their fertility and testicular function. Most insurance companies look favorably upon that–but rarely cover vasectomy reversal or sperm retrieval, which can be very expensive.

[Physician #8]

Providers themselves may understand and accept, rather than challenge these limits. This physician continues:

I’m not at all convinced they should cover vasectomy reversals, but primary infertility is not the patient’s fault. If you volunteer to have a vasectomy, you need to pay the consequences of having it reversed.

[Physician #8]

Insurance companies may also simply not want to commit to coverage in advance, and instead review each claim on a case-by-case basis, making the process variable and unpredictable between patients. As a female patient, working in an office, said,

My new insurers told me to send a pre-authorization. So, I’m not sure right now if they’re going to cover it or not. I’ve talked to other moms. Some of them have gotten their insurance companies to cover it, some haven’t.

[Patient #3]

Still, the precise definitions of the criteria used by insurers may appear ambiguous or arbitrary. Partly for legal reasons, insurers may not want to make such promises up front or in writing.

We were told they’ll never send a letter saying that they will definitely cover it. If your husband gets laid off or quits his job, and you have a letter saying they are covering this, they have to cover it—whether he’s still employed there and paying his insurance or not.

[Patient #4]

Legalistic impediments may also make coverage murky. Insurance companies may erect logistical obstacles as well, including lots of vexing fine print. The fact that policies may vary between states can also create impediments. One woman, who stopped working to attend graduate school, moved; but her insurance policy remained in her prior state. (“They say, ‘The policy was written in another state, so we don’t have to cover it.’ I’m going to have to pay cash.” [Patient #1])

Patients may struggle to communicate with the bureaucracies’ large corporations seeking answers, but in vain. This patient continued,

I printed out all the stuff that I found on the insurance commissioner’s board and gave it to the lady who does the insurance and both fertility doctors. I guess she’s looking into it. I don’t know who else to tell. I guess I can call the insurance commissioner and ask them some questions about how we go around this.

[Patient #1]

Patients have to confront and grapple with these obstacles in addition to other stresses.

Even after the procedure, patients may remain unsure whether their insurer will cover these expenses. These insurance stresses can be the hardest aspect of the treatment process.

It’s a hard, long process—the crappiest. Keeping really good notes of who you talk to, what they say, helped. I was lucky [not to have any symptoms]. I wasn’t tired, so for me it wasn’t terrible, but it certainly could have been a lot easier and a lot shorter time—not 6–8 months to cover a claim.

[Patient #4]

#### Retrospective regrets

Due to limitations in resources and insurance, many patients feel, in hindsight, that they should have selected infertility treatments differently. Depleting their insurance coverage and/or other financial resources, and knowing what they know now, patients who have not yet had children often rue their earlier choices.

Had I known in the beginning, I probably would have figured out a way to make my health benefits go further—gotten three rounds of injections, instead of two. But it’s too late now. The biggest problem for women is finding the money for it. You learn as you go.

[Other Provider-patient #8]

In hindsight, many patients feel they did not budget their limited resources well. Many patients do not fully grasp at the outset the implications of limitations on their insurance, such as possible restrictions on their life-time total coverage, making optimal planning difficult, if not impossible. Costs can also mount more than expected or planned, and patients’ finances can rise or fall.

We considered IVF three years ago, but couldn’t afford it. So we checked into reversing my vasectomy. I looked online. There were some issues whether it would really work. A urologist said, “I’ve done many, even older than yours, and they work.” I’d already been 15 years from my vasectomy. I told her: “Just fix that.” It was only supposed to cost about 10 grand, but ended up a little over 13. But here I am working on my third year and still nothing!

[Patient #2]

Patients may plan for certain costs, but the eventual procedures can cost even more.

Subsequent providers may also denigrate prior physicians’ treatment decisions, if they proved not to work. The patient above who mistakenly thought a vasectomy reversal would resolve his infertility reported that a subsequent doctor said the expense was a “waste.”

On the Internet, I found a doctor who does IVF—two cycles for $13,500. He said we “wasted our time and money doing the reversal after 15 years.” It sounds like the doctor “just wanted to make some money off of you.” You could have spent the same money on IVF and would already have had a kid.”

[Patient #2]

Many patients feel they might have instead shopped around for less expensive providers, but were constrained from doing so. As one health care provider who sought treatment, said:

The hospital where I work drills in our head, “Anything that you can do at our hospital, has to get done here. Otherwise we don’t cover it.” They charge $1,450 for a hysterosalpingogram. But one doctor said, “I could have sent you somewhere else for only $350!” So that ate up an additional $1,450, rather than $350. I could have used that thousand dollars for a third try.

[Other Provider-patient #8]

Patients may find out only afterwards that doctors’ fees vary.

#### Factors exacerbating stresses of uncertainty

Several factors, particularly the fact that patients want only full, not partial success, heighten these difficulties. Patients seek only one goal—a take home baby—not pregnancy alone. But that goal can be hard to achieve. For other medical conditions, such as chronic disease, treatments can yield partial cures for which patients are grateful, even if constituting merely momentary stays against eventual disease progression. But for infertility, such partial success is wholly unacceptable. Patients understandably see a pregnancy that results in a miscarriage as a failure.

The fact that for many women, time is limited, further exacerbates these financial stresses. Not only might resources be limited, but many patients feel they are racing against the biological clock. (“How can I take four months and save up money? I’m freaking out because I just turned 44. My biological time clock is ticking.” [Other Provider-patient #8])

### Patients’ responses to limited resources and insurance

Limited insurance and resources can profoundly shape patients’ treatment decisions in several ways.

#### Delaying, saving money, or saying no

Patients must balance insurance costs, coverage, and resources against likelihoods of success; and may forgo treatment or postpone and try to save money for it. “We didn’t realize it was going to cost us a couple of thousand dollars. We waited a little while and tried to save the money.” [Patient #1]) Only afterwards, in retrospect, will patients know if these decisions turn out to be good or bad.

#### Going to other jurisdictions for treatment

To obtain treatment and overcome barriers posed by geographic and other factors, many patients resort to a variety of strategies. Patients even move their homes—to states with mandates for IVF coverage.

Yet moving locations because of IVF is stressful, inviting significant tradeoffs. One female office-worker, who relocated to another state in order to obtain insurance coverage, said,

The most difficult decision was moving. I didn’t really want to move. I liked our apartment, our friends. We were closer to my family. It was a little traumatic. That was hard.

[Patient #7]

The cost savings can, however, be considerable, and thus perceived as worth it, especially for patients with fewer resources. She continued,

We had no desire to leave. But when we looked into it, the options were: It’s going to cost $20,000 a year per cycle here. If we moved there, insurance would cover it. So we moved.

[Patient #7]

Still, even after relocating to a different state with mandated coverage, and receiving fertility treatment, patients do not always get pregnant, which can be crushing.

When the two cycles didn’t work out, we were devastated, because we had moved there, and didn’t really like it. We had shifted our whole life for a baby, and it wasn’t even working. I gave everything up, and still had nothing. We were so stressed. I had to wear a mouth guard at night because I was grinding my teeth and my jaw was starting to hurt so badly. When you try naturally, it’s very disappointing every month when it doesn’t work. “But when you’re going through IVF/PGD, you never feel yourself, or can tune it out at all. It’s intense.

[Patient #7]

She thus moved overseas, and only then got pregnant.

So we decided to move abroad. We had always talked about living in Europe for a while. My husband was able to get some consulting work there. That was when I ended up getting pregnant naturally!

[Patient #7]

Limitations in insurance coverage can thus foster extended geographic, as well as medical, emotional and financial journeys.

To obtain better insurance, patients may also consider or pursue changing jobs.

My husband thought of looking for a different job—just to get better insurance. But it’s not easy to look for a job based on the insurance coverage; because they don’t give you all the details in the New Employee Handbook.

[Patient #1]

Patients may also change their insurance over time, but to little or no avail. (“We went back to him when my husband had a new job, so we had better insurance, but the insurance still wouldn’t cover it.” [Patient #1])

Yet while some patients feel able to move or change jobs in order to obtain IVF coverage, many others feel stuck in locations or employment that they don’t like, but that provide for a degree of IVF insurance coverage. Still other patients or their families may all lose their jobs, or their insurance company may change, altering fertility plans:

The insurance I had before covered the testing; but I lost that job. So my husband was trying to find better insurance. The job he has now has a different insurance company, but there is no fertility coverage at all.

[Patient #1]

Patients may even consider seeking treatment in the developing world, given cheaper treatment there, though often fearing the quality of such services.

You can go to India and get it done for a third of the cost, but that doesn’t sound safe. And travel there would be expensive. I think about some guy sitting in a back room in a little hut somewhere with a dirty knife. You’re in their country under their rules and regulations, with no protection or safety that we have here. And the expense of getting there—the plane tickets, and need to stay for a period of time. You can’t have it done that night and come home.

[Patient #2]

#### Searches for free and discounted treatments

Many patients seek free consultations and treatment, often advertised online or elsewhere; but these usually prove limited or illusory.

A lot of clinics offer free phone consultations. That was really helpful: having a free 20-minute, and then another 30-minute consultation. There’s a program that helps pay for the medicines, but unfortunately has income qualifications: you have to be poor. They go by the poverty guidelines. I have yet to submit my paperwork, because I was waiting to see if my third IUI would have good results.

[Other Provider-patient #8]

Many clinics offer various “all inclusive” treatment packages. Ultimately, however, these bundled packages may still cost more than prospective parents can afford. Even a package that includes a money-back guarantee can nonetheless constitute a “gamble.” Another female patient, working in an office, said,

A pre-pay IVF company would charge me $45,000 upfront for three IVF cycles, based on my factors. If it didn’t work, I would get a portion of that money back. But I was not willing to make that kind of investment.

[Patient #5]

“Bundle packages” offered by several clinics attract many patients, but can nonetheless also have hidden added costs and obstacles.

I went to a different doctor who offered a bundle package: $985, no matter how many times you had to go in. That was nice. But the medicines were not part of the package!

[Other Provider-patient #15]

Many patients search, too, for inexpensive alternatives—research studies, and grants. But these options may prove problematic as well.

We’re looking for some programs. One place has studies, and we put in for one. Sometimes the government funds studies: if you’re selected, everything will be paid for—except for the retrieval, and any medications, which our insurance would pick up. So, we applied a few months ago, but haven’t heard back. We’ve called around, checked other states to see if there’s other inexpensive ways.

[Patient #2]

This patient hopes to do IVF, but remains highly uncertain whether he will be able to afford it—let alone what the outcome will be.

Several patients would like a website that lists free resources and services, but these may be elusive. “A website how to obtain assistance would be totally awesome. I’m looking for assistance—help with the medicine, or subsidy programs, or something.” [Other Provider-patient #8]

#### Misleading offers for free IVF

Many patients search online for free IVF clinics and services, but these generally prove misleading, too. Online links advertise free services, that often turn out to be elusive. “You get a lot of empty promises out there. One site says ‘free IVF.’ But when you get in and start looking into it, it doesn’t mention anything about it being free.” [Patient #2] Many websites are hard to evaluate and may simply want your money.

A lot of bogus stuff is out there. It’s hard to find the stuff that isn’t making a dollar off you. All that they’re looking for is “put your credit card number here.” Some sites ask for information, and want you to pay, teaching you how to find the stuff: “Pay us $49.95, and we’ll show you how to get the money to have your IVF done—raising money through your family, or going abroad and having it done…” There’s always someone trying to separate you from your dollar. That’s the American way.

[Patient #2]

Apparent grant-givers often require a lot of personal financial information, raising suspicions.

Some of the funding out there wants you to send them two, three years of your tax returns, how much money you have in your checking account, just to get a $10,000 grant. That’s too much information. They call them scholarships for IVF. You have to open up your life for them. It looks like a legit site, but they want a donation of $25 or $250 a month. If I had $25 or $250 a month, I could just pay for my own treatments. So I don’t see why they call it free…They say they help dreams come true, but they’re only going to give you about $10,000 if you get selected…There’s always a loophole.

[Patient #2]

Some clinics claim to offer money-back guarantees. They have programs. (“If we can’t get you pregnant after three cycles, here’s 80% of your money back.” [Patient #2]) But not all pregnancies result in babies.

#### Using other women’s leftover drugs

Providers and patients have at times sold one patient’s leftover medications to aid other patients, but problems can ensue. “People donate drugs back and discreetly give them to help these women.” [Other Provider #6]

Women have also created black markets on the Internet to buy and sell unused ART medicines at a discount. Yet dangers can lurk.

I searched “free Bravelle,” and “free vials of Bravelle” to see if there were any programs, or pharmaceutical company contacts, and if I could get some. Maybe doctors’ offices have free samples in their cabinets. I’m desperate. I found a black market website where a lot of women sell leftovers at a much cheaper price. That’ll probably end up being my next route. It seems okay. Women post their leftovers. Usually with a little story: “We’ve stopped trying now, and are adopting.” I just hope they’re not putting baby powder in those bottles!

[Other Provider-patient #8]

As she suggests, however, black market purchases can pose safety risks.

#### Requests to alter billing

Patients may ask providers to lie on their behalf to insurance companies—to list other reimbursable indications for procedures that were performed. Understandably, these clinicians may decline.

The billing department said that if I could have the hysterosalpingogram resubmitted under a different code—like endometriosis, rather than infertility, they would be glad to resubmit everything and it would be a piece of cake. But my doctor’s not willing to do that. She’s scared about malpractice, etc. But I could have been reimbursed for the whole $1,450.

[Other Provider-patient #8]

### Providers’ Responses

Current policies also pose stresses for providers, who can play key roles in these patients’ struggles, but range widely—from seeking to reduce these tensions to inadvertently increasing them. When a patient’s insurance coverage ends, a few rare providers have continued to offer treatment for a while at a lower fee. “Doctors try to help out their patients and give them discounts if it’s been a few cycles.” [Other Provider #3] But overall, that appeared rarely.

Some physicians have also given patients free drug samples. Yet free medications, even if provided, may not be the most affective.

My first two IUIs were done with injectables, which have much better results. The third one used a drug that the doctor had a free online coupon for. She said she would try to keep stashing samples of drugs. But I don’t know how many samples she can get for me. It’s a money factor.

[Other Provider-patient #8]

Providers may not all be as sensitive as they might be to these difficulties. Patients felt that, despite the uncertainties involved, certain providers were overly optimistic that their treatment plans would succeed, sparking patient frustration, anger and discontent if these procedures then failed. Patients with limited resources may, in retrospect, perceive providers’ therapeutic optimism, which, when it proves wrong, can be especially frustrating. When a costly procedure fails, despite prior assurance from the provider, patients may feel angry, misled, and even deceived. Patients may like providers’ initial optimism, even if suspecting that treatment might fail, but nonetheless get swayed by the provider’s hope. (“I shook his hand when I left, understanding he can get it done. But I now feel like he gave me false hope.” [Patient #2])

Patients look for assurance (e.g., through handshakes), but it can prove unreliable or elusive. Providers may also vary in how much they appreciate and respond to patients’ financial limitations.

### How much is a child worth?: The stresses of making these decisions

Current policies also force patients with ongoing failures to have a child to wrestle with the ultimate excruciating ethical dilemmas of how much a baby is worth—exactly how far to pursue treatment. “Prospective parents confront terrible dilemmas: how much money it is worth to have a child” [Patient #5]–at root, putting a price tag on creating a child.

These dilemmas are especially difficult, since a child can provide deep meaning, and for some individuals, the value of bringing one’s own child into the world is seemingly priceless—worth enormous sums. Prospective parents may see having a child as more valuable than even their own lives.

While pregnant, I was diagnosed with thyroid cancer. They tested a nodule and said, “We’re 99% sure it’s cancerous.” But I was more nervous waiting for my IVF cycle results than getting that biopsy report! If somebody had said to me, “This might kill you, but might work in getting you pregnant,” I would have done it! It was more valuable than my life!

[Patient #7]

Many couples who succeed in having children thus feel that in the end these costs are justified.

One couple I know spent $30,000 on and had a baby girl. They thought it was the greatest thing. They couldn’t be happier with anything in the world, but are now burdened by debt. But to them, every penny was worth it.

[Patient #2]

Yet conversely, since having a child can fulfill an ultimate lifelong dream, costly unsuccessful efforts can represent exasperating ordeals that outsiders may not fully appreciate.

People who haven’t gone through it don’t understand. I never aspired to be a doctor, lawyer or teacher. My only dream was motherhood. So people say: go for it at all costs! Unfortunately, that’s not reality.”

[Patient #5]

Many prospective parents go to enormous lengths, making major sacrifices to achieve the goal of having a child, confronting and making difficult trade-offs concerning these expenses. At multiple points, many prospective parents have to decide whether to continue to pursue this goal or stop—reevaluating their goal. They respond in various ways. As one nurse said,

I’m already working every single day of the week, working doubles at both hospitals, 18-hour days, sleeping for three hours, going back to work at the next hospital, earning as much money as I can. I can scrounge up enough, the thousand extra dollars a month, just to do an IUI, but not enough for the medicine.

[Other Provider-patient #8]

Patients may end up using funds for IVF, rather than for other important expenses, such as repaying student loans—sacrificing and/or forfeiting other opportunities in order to pursue IVF.

I could probably be using this money to pay off student loans, so I wouldn’t have so much debt later. To get a doctorate in graduate school is going to be over $100,000, and I’ve still got undergraduate loans!

[Patient #1]

Patients may use present or future savings or other income that they might otherwise have devoted to other long-term goals. Yet uncertainties may linger, too, concerning future income and expenses.

I’m banking on an inheritance my uncle left me, which I’m hoping comes by August. If that’s the case, I’m going to take the 10 grand, and do IVF. My cousin, who’s handling the affairs, says it should be coming. But I can’t bank on that.

[Other Provider-patient #8]

Patients may go into substantial debt, deferring payments. “A friend pregnant with twins put her IVF on a credit card. I thought that was ridiculous.” [Patient #5]

Given these financial as well as medical uncertainties, patients must make difficult cost/benefit analyses and comparisons. Patients wrestling with how much to spend, and what approaches to use may see these costs as investments that may or may not succeed, and many individuals come to draw sharp lines, beyond which they feel the price is too dear. Still, patients vary widely on where they draw this line, struggling to weigh the costs against the odds of success of various approaches, making complex assessments due to combinations of finances and desires. Many individuals proceed to pay large amounts while others can’t or won’t. The most difficult choice can be whether to do IVF vs. adoption or other procedures, each of which has varying pros and cons that can be difficult to compare.

Deciding whether I wanted to go through one more procedure in order to try and carry a child was really difficult. The hardest part was whether or not to do IVF.

IVF is a huge cost. I never totaled it up because I didn’t want to know. But I would say $16,000 –vs. adoption. Each round of a procedure represents a gamble, yet the odds involved are hard to assess. Based on my history and age, my doctor gave me a 40% chance of the IVF being successful. So we had to consider: Or do we want to spend $16,000 on a 40% chance? Do we want to spend $25,000 on the chance of adoption? I didn’t know where I should invest the money: put $16,000 into a 40% chance? Domestic adoption would cost between $25,000 and $40,000, and the birth mother can change her mind. Then, there are all the options in between: donor eggs, surrogates. It came down to a financial investment—your cost-analysis and the odds that your doctor gives you.

[Patient #5]

Patients range widely in how they view and weigh these tradeoffs, and make these decisions. In the end, emotional factors may profoundly determine these choices.

With IVF, I knew all the emotional components: I’d be prepared. If we put this investment into adoption, I don’t know what to expect. By the time we got to IVF, I was emotionally drained. I thought, “At least I’m familiar with this emotional set.”

[Patient #5]

Given the multiple uncertainties involved, input from friends and family members can itself conflict.

Everybody around me said that we should start with IVF. I said, “You’re crazy if you think we’re going to spend $15,000 for a 50/50 chance. So we started with IUIs. In retrospect, I wish we had gone with IVF first. But we didn’t because of the costs. I say “If your insurance will cover it, go for IVF first.” You’re giving yourself injections anyway. You have the same emotional investment, and if your insurance covers it, why not go where your odds are the highest?

[Patient #5]

Over time, as patients reevaluate their thresholds concerning the costs and odds of success, their perspectives, and the amounts they are willing to spend, can fluctuate.

I drew lines, erased them, drew them again, erased them and drew them yet again. If you asked me at the start, how many rounds of IUIs I would do, I would have said one or two. I wound up doing six! I wasn’t willing to go into complete debt, to take a second mortgage on my home, to get so far behind that by the time we had a child, we had nothing for that child. We joke that when the kids ask for a bicycle, we’ll say, “Too bad. You were born!” We can still provide for them pretty nicely. Luckily we had a bit of help from my family, and took advantage of a 0% interest credit card.

[Patient #5]

#### Finances causing marital stresses

Not surprisingly, members of a couple can disagree over these issues—how much a child is worth to each of them. Individuals must thus face questions of not only whether they want a child, but how much they are willing to spend more than they otherwise want to accommodate their spouses’ wish to spend more. One member of a couple, but not the other, may want to go to extreme measures, causing personal and marital strains.

Some people will do anything and not stop: Take out loans, do strange things. One man’s wife was putting a lot of pressure on him to sell his Harley Davidson motorcycle. He thought long and hard: This isn’t worth it. She isn’t worth it. I thought he was going to break down in my office! People have different values.

[Physician #8]

These financial burdens can significantly strain a relationship in multiple ways. “It puts a hardship on married life, home life. The number one thing that will tear up a marriage in a heartbeat: finances.” [Patient #2] Yet providers may not all grasp or appreciate the extent of these phenomena.

### Implications

#### Changing policy?

These cost constraints raise larger public policy questions as well. When asked if they thought more regulations might be needed, most providers and patients argued for government policies to mandate or increase insurance coverage, but were wary of other government regulations concerning ART. Heightened insurance coverage could aid not only patient finances, but yield other benefits as well, such as reducing an incentive for twin or higher multiple births. Due to finances, many patients who want eventually to have more than one child will seek to have twins, rather than a singleton pregnancy, even though twins have higher rates of medical complications [41].

Legislation feels dangerous. But if insurance were available, people would take fewer risks in terms of the number of embryos transferred. I have seen disasters, with being pregnant with a higher order of fetuses.

[Other Provider #4]

Several patients think government money spent on other programs can be better used for ART. (“A lot of money is wasted on other programs—a lot of welfare moneys are unnecessarily.” [Patient #2])

Yet patients and providers were frequently aware that if the government or private insurers ended up covering more IVF expenses, someone would ultimately have to bear the costs. (“I wish the insurance companies would cover it, but someone would have to pay for that too.” [Patient #2])

Nonetheless, most patients and providers felt that coverage for infertility should be mandated, feeling that it is for serious disease.

It should be part of the health plan. If you have a life-threatening disease, it’s covered. If you have infertility, it may or may not be covered. That doesn’t seem fair.

[Patient #7]

Providers tended to be more aware than patients of additional specific challenges that existed to government mandates to increase coverage. Mandated coverage can help many patients, but poses conundrums given limited resources, rising health care costs overall, and competing public health needs, of how much to provide to everyone, and who should receive coverage—whether insurance should cover only patients with medical causes of infertility, or single and gay and lesbian patients as well.

Mandatory infertility coverage for everybody can make the system better so they don’t have to make decisions for financial reasons and put in 3 embryos when they don’t have money to do another cycle. Unfortunately, money drives a lot of this, as well as changing social mores. The world is changing. It doesn’t have to be Ozzie and Harriet anymore. Families can be put together in different ways. Still, we shouldn’t be squandering our resources. We need reasonable limits.

[Other Provider #3]

But clearly tensions persist, concerning who should draw those limits, and where, and what kind of evaluative process to use. “There’s got to be a limit in the number of children. Two children per family is reasonable (not like [certain groups] with 18). That could be federally mandated.” [Other Provider #6]

Nonetheless, many providers are not optimistic about insurance coverage increasing anytime soon.

I don’t think there’s any hope of changing insurance in the immediate future. To mandate would not be very in favor politically. The 9 or 10% of people with significant infertility would be for it. The rest?

[Physician #10]

In part, infertility treatment may be considered elective—unlike procedures in other medical specialties that aim to eliminate life-threatening disease. Here, procedures may all be considered elective. “It’s like cosmetic surgery.” [Other Provider-patient #9]

Many providers assumed that insurers may also not see childlessness as a priority or relatively important problem.

They don’t look at childbearing as a health issue. If a health plan has so much money, how far down the list are you going to fund? They draw the line, and can’t fund anything below that line. Preventative care for pregnant women, and treating all the diabetics and related diseases, ends up on top of the list. The fact that somebody can’t have a baby is lower on the list.

[Physician #5]

## Conclusions

These data, the first to explore how providers and patients view, respond, and make decisions regarding limited insurance and resources for ART, raise several critical ethical and policy concerns. Current policies force many patients and providers to struggle with complex medical, financial and ethical strains regarding the cause, length, cost and outcome of infertility treatment and the odds that insurers will cover these expenses. Most of these patients were employed full-time and had a certain amount of resources or insurance for infertility treatment, but these amounts ultimately proved limited, forcing difficult choices.

These data suggest several key findings that have not previously reported in the literature. Firstly, insurers cause stress because of not only what they decline to cover [10], but how they make and communicate these decisions. Commonly, insurers will not commit to coverage in advance, and instead decide only afterwards, and on case-by-case bases. Consequentially, patients must often pay first, and later struggle to get reimbursed, which can take months. These data highlight how patients and providers then face uncertainties, unable to predict in advance how best to allocate limited coverage amounts, causing strain. Secondly, while prior data have suggested that costs cause anxiety and lead many patients to stop treatments or rely on savings [6, 7, 10], the current data suggest how patients may respond in other ways as well, adopting several strategies—e.g., moving and/or seeking treatment in other states or countries, switching or feeling “stuck” in certain jobs because of insurance coverage, seeking “free” treatment online, or “free” medications through providers, the Internet or the “black market” or making other financial sacrifices, or altering other goals or life plans—e.g., using funds they had specifically intended for other purposes such as going to graduate school.

Thirdly, while the prior literature has examined how women may feel stress and stop treatment because of expenses, the present data highlight how members of a couple often make these decisions jointly, but can disagree, impacting relationships. Fourthly, while prior research has shown that limited insurance coverage impedes many patients from seeking IVF treatment [6–8], the present data suggest that patients do not perceive and respond to resource limitations as fixed phenomena—i.e., patients do not see treatment simply as either “affordable” or not. Rather, patients who have not yet succeeded in having a child often face quandaries of how much to keep spending—how much a child is worth—and are forced to make complex risk/benefit calculations. Patients may face a complex series of quandaries and vagaries, spanning a wide spectrum, and ranging in how far they go, having to choose exactly how much to spend, whether to incur debt, and if so, how much, and frequently continue to reevaluating these decisions over time, shifting when treatments fail to yield a ‘take home baby.’ Many patients debate whether and how much to go into debt. Fifthly, while prior research has suggested that costs affect patient decision-making [6, 7] and cause anxiety [10], the present data highlight how these effects are in fact worsened, exacerbated by other factors and strains patients confront. Patients are often racing against a “biological clock” and seek only a “take home baby,” not pregnancy alone, thereby struggling with difficult and nuanced tradeoffs. Sixthly, while prior data have documented that patients are concerned about costs [4–7], the present data suggests how providers also view and respond to these challenges, but vary in doing so, and in their sensitivity toward these patient difficulties—from advocating for patients to insurers, to offering overly high expectations for success, and criticizing colleagues’ prior approaches. Though these clinicians may not have actually wholly fit these descriptions, patients felt that these providers did so, which is itself worth noting.

Seventhly, these data suggest that, since most IVF cycles do not produce a “take home baby”, a feedback loop exists, with many patients repeatedly having to reconsider how much to spend, and whether to seek more treatment, or adopt, or not have a child. These data thus indicate how choices about paying for infertility treatment are commonly not one-time decisions, and how several specific variables, related to numbers of past failures, resources and opportunity costs, can shift over time.

Eighthly, these findings shed light on ways of developing a rational choice model concerning selection of possible approaches in infertility treatment, but also several challenges in doing so. While rational choice modeling has been applied to decisions of whether to transfer one or more than one embryo [22], the risks, benefits, and criteria in that choice are more strictly biological and clearly characterizable than in decisions here of whether to pursue having a child and/or a fully biological child (rather than one produced from the gametes from none or only one of the prospective parents). The health belief model, applied to many areas of patient decision making, similarly theorizes, in part, that patients weigh the risks and benefits of a particular procedure [42].

These data provide vital insights for the development of rational choice models, identifying, clarifying and framing specific decision points concerning various treatment options and criteria. Future research and efforts to develop and test how such models might focus on just one or more of the decisions in these multi-stage journeys––e.g., how patients in fact weigh these considerations, and change after each successive treatment failure, how members of a couple who disagree on how much to spend end up negotiating differences, and/or change over time, how insurers make their decisions, and how providers decide how to advise and/or consult with patients about these issues over time.

Yet the present data also underscore how patients may confront multiple complex economic, social, psychological and ethical decisions, involving deeply personal, subjective dreams and values, made in the contexts of intricate relationships among individual members of couples, and with providers, insurers and others, and potentially shifting over time. Quantifying the significance and worth of a future human being’s life to childless potential parents, translating these profoundly life-altering, deeply personal quests for fulfillment into numeric values or utilities as economic “goods”, can be extremely hard for members of a couple, both individually and together. Creating a child can have profound psychological, social, cultural, moral, and even religious value, providing ultimate personal meaning and purpose that is not wholly measurable. Individuals seek sources of symbolic immortality—connections to provide senses of ultimate meaning, often through offspring or religion [43]. Arguably, for many individuals, in no other area of economic life is the potential value of the goal as high. Indeed, anecdotally countless parents sacrifice major aspects of their own life for their offspring, with some even valuing their children’s lives more than their own, highlighting the limits of economic and other rational decision models in this realm.

While prior critiques of rational decision-making models have described how the amount of available information is often restricted, that rationality is thus “bounded” [44] and that individuals often weigh losses more than gains [45], the present data suggest that challenges occur, too, because individuals, desperate to have a child, may have difficulty translating these personal meanings into wholly economic terms. Moreover, the causes of a patient’s infertility may be unclear, due to limited scientific understanding. Relevant and reliable data on outcomes for particular patients can be lacking and/or hard to obtain. Doctors may provide overly optimistic estimates (e.g., “false hopes”), and eager patients may minimize or deny age-related decrements in infertility. Insurance companies may fail to communicate in advance whether they will reimburse costs, and if so, how much, and based on what criteria. Moreover, individuals are frequently inaccurate in “affective forecasting”–correctly anticipating their emotional reactions to future major events[46]. Individuals’ preferences may also change over time when initial assumptions and expectations about cost-benefit decisions prove wrong due to the complex medical, financial, social, and psychological uncertainties involved.

Yet though standard models of rational decision-making thus face considerable challenges and may apply here only with difficulty, these data suggest ways, as discussed above, of helping to develop such models as much as possible—by trying to quantify these various specific types of uncertainty and incorporate them into a model.

These data have several critical implications for future policy, practice, research and education. These data, illuminating the impact on patients’ lives of current government and insurance company policies regarding ART, can inform ongoing policy discussions and debates—e.g., about whether states should mandate that insurers cover more infertility treatment. Specifically, these interviewees highlight stresses and other consequences of current limitations that have not heretofore been empirically probed, concerning not only what insurance companies decide, but how they do so and communicate these decisions to patients. Efforts to increase insurance coverage generally should continue, but hurtles remain, and countless patients will doubtlessly continue to confront limitations. Nonetheless, legislatures and professional and governmental organizations in multiple countries could at least strongly encourage insurers to be more ‘upfront’ and transparent in their coverage—to clarify policies and coverage for individual patients, to help patients determine whether expenses will eventually be covered, and what criteria are involved in coverage decisions, to make coverage less unpredictable. Such organizational entities could issue guidelines that providers make full costs clear to patients, ensuring that patients are aware beforehand of all hidden or excess expenses.

These data also suggest that current policies can foster “black markets” of drugs. These markets may not always be safe, since medications may have expired, or been tampered with. Providers should be as aware as possible of these phenomena in order to caution patients about possible harms or scams. Yet in the US, 36 states have enacted laws for the return, reuse and recycling of prescription drugs, with certain safety precautions generally applying (e.g., drugs must be in sealed packaging and cannot be expired), and 20 of these states have operational programs, saving patients millions of dollars [47]. Patient organizations could potentially assist in establishing legitimate mechanisms for offering patients left-over medications that meet such safety criteria. ASRM or other professional or patient advocacy organizations could also potentially develop guidelines concerning advertisements about IVF treatments, given that Internet searches for “free IVF” reveal multiple sites that may mislead many patients.

Questions arise of whether insurers should cover ART more. Insurance generates stresses for patients in other areas of medicine as well, but does so acutely here. Insurance coverage may be limited partly due to controversies as to whether “infertility” indeed constitutes a disease per se and/or a public health priority. People can live healthy lives without having children, though having offspring can also add much to parents’ lives. Some insurers cover some infertility treatment, but only if medical reasons exist, rejecting patients who have social and structural barriers (e.g., being single and/or gay). Yet, insurers often cover expenses when a “disease” per se is not involved—e.g., contraceptive drugs and devices. These data thus highlight critical questions for insurers, policymakers, scholars and others to explore regarding where and how limits in infertility coverage should be established, given that the status quo causes considerable strain on many patients and providers.

Enhanced education of patients and providers concerning these medical, policy and ethical issues could also be beneficial. Many patients may not optimally plan and prepare for limitations in coverage and resources. Patients may seek to gauge costs involved, but these are often unclear. Patients may thus benefit from assistance in making these complex choices and plans. Crafting and disseminating realistic messages can be helpful—e.g., elucidating patients’ options if they have a particular amount of resources or coverage. Heightened provider awareness of these stresses and complexities can also be beneficial, allowing clinicians to assist patients as much as possible.

Clinicians could seek to ensure that patients are fully aware of the realistic odds of success of any procedure, and of the limitations and uncertainties of costs and insurance. Websites could be developed for patients, regarding needs to be prepared for the unpredictabilities and possible lengthy periods of treatment required, and insurance limitations. Mental health professionals can also assist patients in confronting these quandaries. These data also raise questions about how providers communicate about each other to patients—what clinicians say, and how often they criticize colleagues’ decisions. Professional norm generally impede providers from criticizing each other to patients; but doctors may occasionally do so regarding ARTs—perhaps because ARTs are relatively new, and consensus is hence often lacking concerning standards of care in particular situations, given uncertainties regarding diagnoses and treatment, odds of a birth from any one cycle being < 50%, and patients seeking only complete, not partial, success.

These data also raise questions concerning therapeutic optimism, which contributes to patient misunderstandings in other areas of medicine [48]. With infertility, clinicians’ optimism may also reflect the fact that providers may have conflicting interests, since they are profiting from each procedure. Since ARTs are generally covered by insurance only minimally, if at all, prices may be higher than they would otherwise be and receive less oversight or restraint from insurers or others. Since this market is in many ways unregulated and lacks transparency, professional organizations could potentially urge providers and insurers to publish and clarify, up-front, rates and reimbursement for patients. However, providers and insurers may, for various reasons, resist doing so.

These data also highlight needs for more research concerning ethical and policy dimensions of ARTs, and suggest a research agenda in this area. Prior studies have explored a few aspects of these technologies, but the rapidly increasing variety and use of these technologies pose evolving ethical and policy challenges. Research can assist providers, patients, policy makers and the public more generally in grasping and making appropriate decisions about these realms. Future research can explore further, for instance, using large samples, how and with what frequency patients and providers perceive and confront these difficulties, assess these tradeoffs, make decisions regarding tensions between personal meaning and economic constraints, and how these change with time; how often patients consider or adopt these various strategies and move, switch or stay stuck in locations or jobs because of insurance, and/or go into debt, and for how much; how often full costs are “hidden” or unclear to patients; how, when, and to what degree patients and their spouses disagree, and how they then negotiate these differences; how often providers advocate for patients with insurers, and succeed or fail, and which strategies, if any, succeed more. Longitudinal studies can assess how patients who exhaust all of their resources and/or go into debt look back at their decisions. These data underscore, too, how more research on factors that affect outcomes can aid physicians and patients in estimating more precisely the anticipated type(s) and amount(s) of treatment that a particular patient may need, based not only on population-level epidemiological data, but individual patients’ specific biological and other characteristics.

These data have several potential limitations. The sample size is adequate for qualitative researcher to elucidate the themes and issues that emerge, but not for statistically analyzing how different groups (e.g., male vs. female physicians; or physicians vs. patients) vary; however, future studies can investigate these issues with larger samples. In addition, these providers and patients are from the US, highlighting needs for exploring these issues in other countries—especially where insurance coverage for treatment is limited. Still, clinicians, in particular, are very busy and are increasingly difficult to recruit for research, with response rates decreasing in recent years [49, 50]. Difficulty recruiting larger numbers of providers doubtlessly contributes to the lack of any prior studies on these critical questions, and the value of the current data. Moreover, these data also arguably have a certain face validity, illuminating challenges that many providers confront.

In sum, these data, the first to examine several key aspects of how IVF providers and patients view and make decisions regarding cost and insurance constraints for infertility treatment, raise crucial ethical and policy issues that have key implications for future practice, guidelines, research and education of providers, patients, insurers, policymakers, and others.

## Supporting information

S1 File Semi-structured interview for providers. (DOC)
