# Supplementary material for: How much is a child worth? Providers’ and patients’ views and responses concerning ethical and policy challenges in paying for ART
Source: PLoS One. 2017 Feb 16;12(2):e0171939. doi: 10.1371/journal.pone.0171939 (PMC5313146; doi:10.1371/journal.pone.0171939)
Supplement: S1 File — (DOC) [file pone.0171939.s001.doc]

## **SEMI-STRUCTURED INTERVIEW FOR PROVIDERS**

**Background information:**

- How would you describe your practice, and your patient mix (i.e., by types of procedures, ages, diseases, insurance coverage, ethnicity, and religious beliefs).
- Approximately how many patients do you see per year?
- Do you have a private or hospital based practice?

**General:**

- How do you view current uses of IVF and PGD?
- What challenges do you face in your work as an ART provider?
  - How do you address these challenges?

**Specific procedures:**

- PGD:
  - Have you ever recommended PGD/PGS to patients? For what indications? How often? How did you make these decisions?
  - Have you ever decided not to support use of PGD/PGS? Why?
  - Do you have any concerns about patients using PGD/PGS? If so, what?
  - Have any changes occurred over time in your PGD/PGS use? If so, what?
  - What obstacles exist in patients’ use of IVF/PGD? Have you tried addressing these? If so, how? With what effect?
    - What are the most difficult IVF and/or PGD decisions you have faced? How did you resolve these?
    - What technical barriers to IVF and PGD exist?
    - What are your PGD and IVF success rates?
    - Have you had any adverse outcomes associated with IVF or PGD?
  - Have you faced challenges or concerns regarding PGD? Non-disclosing PGD? PGD for HLA-typing?
- Other procedures**:**
  - Have you faced challenges concerning procuring oocytes from donors? If so, when? What happened?
  - Have you ever faced challenges concerning egg donor agencies? If so, when? What happened?
  - Have you faced challenges concerning numbers of embryos transferred? Pregnancy reduction? If so, when? What happened?
  - Have you ever faced challenges related to other procedures? If so, what happened?

**Doctor-patient relationships:**

- - Have patients ever been uncertain or confused about using IVF and/or PGD, and if so, how?
- Have you seen any problems in provider or patient expectations and misunderstandings about IVF and/or PGD? If so, what?
- Have you ever faced challenges regarding referrals of ART patients for treatment, patients changing doctors or doctor shopping? If so, when? What happened?
- Have you ever faced challenges related to other aspects of doctor-patient relationships? If so, what?

**Patients’ characteristics:**

- Have you faced other challenges or concerns regarding patients? If so, what? Concerning ages of patients? Non-traditional combinations of parents? Patients’ future parenting abilities? If so, when and how? What has been difficult about these situations? What did you do? How did you make these decisions?
- Have you ever considered not treating a patient? Why? What did you do?
- How have your patients viewed these issues?

**Factors:**

- Financial:
  - - Have cost issues arisen with patients’ use of IVF/PGD? If so, how?
    - Have you perceived financial barriers in IVF/PGD use? If so, what? How frequently?
    - What have been your experiences with patients using insurance for PGD? When have insurance companies agreed or not agreed to pay for IVF and/or PGD? Have you tried to address these issues? If so, how?
  - Institutional:
    - Does the clinical setting in which you work affect your use of IVF or PGD? If so, how?
- Facilitators:
  - - Have any approaches helped to overcome barriers to use that may exist?
    - Do you perceive any facilitators to use of IVF/PGD? If so, what (e.g., informational, financial, attitudinal, institutional)?
  - Personal factors:
    - Have you or anyone you have known well ever considered or undergone IVF? When and why? What were their experiences like? Did that influence you at all? If so, how?
    - Do you have religious, spiritual or other beliefs that affect your views or approaches to IVF or PGD in any way? If so, how?

**Implications:**

- Do you see areas for improving treatment? If so, what?
  - In what areas, if any, do you think additional professional or public education would be helpful?
  - What additional guidance, if any, do you think would be helpful?
  - Do you have other thoughts on these issues?
